# Hippocampal TNFα Signaling Contributes to Seizure Generation in an Infection-Induced Mouse Model of Limbic Epilepsy

**DOI:** 10.1523/ENEURO.0105-17.2017

**Published:** 2017-05-09

**Authors:** Dipan C. Patel, Glenna Wallis, E. Jill Dahle, Pallavi B. McElroy, Kyle E. Thomson, Raymond J. Tesi, David E. Szymkowski, Peter J. West, Roy M. Smeal, Manisha Patel, Robert S. Fujinami, H. Steve White, Karen S. Wilcox

**Affiliations:** 1 Department of Pharmacology and Toxicology, University of Utah, Salt Lake City, UT 84112; 2 Anticonvulsant Drug Development Program, University of Utah, Salt Lake City, UT 84112; 3 Department of Pharmaceutical Sciences, University of Colorado, Aurora, CO 80045; 4 INmune Bio, Seattle, WA 98117; 5 Xencor Inc., Monrovia, CA 91016; 6 Department of Pathology, University of Utah, Salt Lake City, UT 84112

**Keywords:** AMPAR, inflammation, TMEV, TNFα, TNFR, XPro1595

## Abstract

Central nervous system infection can induce epilepsy that is often refractory to established antiseizure drugs. Previous studies in the Theiler’s murine encephalomyelitis virus (TMEV)-induced mouse model of limbic epilepsy have demonstrated the importance of inflammation, especially that mediated by tumor necrosis factor-α (TNFα), in the development of acute seizures. TNFα modulates glutamate receptor trafficking via TNF receptor 1 (TNFR1) to cause increased excitatory synaptic transmission. Therefore, we hypothesized that an increase in TNFα signaling after TMEV infection might contribute to acute seizures. We found a significant increase in both mRNA and protein levels of TNFα and the protein expression ratio of TNF receptors (TNFR1:TNFR2) in the hippocampus, a brain region most likely involved in seizure initiation, after TMEV infection, which suggests that TNFα signaling, predominantly through TNFR1, may contribute to limbic hyperexcitability. An increase in hippocampal cell-surface glutamate receptor expression was also observed during acute seizures. Although pharmacological inhibition of TNFR1-mediated signaling had no effect on acute seizures, several lines of genetically modified animals deficient in either TNFα or TNFRs had robust changes in seizure incidence and severity after TMEV infection. TNFR2^–/–^ mice were highly susceptible to developing acute seizures, suggesting that TNFR2-mediated signaling may provide beneficial effects during the acute seizure period. Taken together, the present results suggest that inflammation in the hippocampus, caused predominantly by TNFα signaling, contributes to hyperexcitability and acute seizures after TMEV infection. Pharmacotherapies designed to suppress TNFR1-mediated or augment TNFR2-mediated effects of TNFα may provide antiseizure and disease-modifying effects after central nervous system infection.

## Significance Statement

CNS infection is a significant etiology for the development of acquired epilepsy. Infection-induced uncontrolled inflammatory reaction in the brain can damage the parenchyma and contribute to acute seizures and the development of epilepsy. A large number of patients suffering from infection-induced epilepsy are pharmacoresistant to available antiseizure drugs. In the present study, we report that elevated levels of TNFα, a key inflammatory cytokine, in the hippocampus after TMEV infection may mediate the excitotoxic effects through the TNFR1-mediated pathway and contribute to acute seizures. Our results suggest that selective inhibition of TNFα-TNFR1 signaling may provide a new strategy to decrease acute seizures and potentially suppress the development of epilepsy.

## Introduction

CNS infection-induced encephalitis is often associated with the occurrence of acute seizures and a dramatically increased probability of the development of acquired epilepsy (Misra et al., 2008; Vezzani et al., 2016). Investigating the role of inflammation as a consequence of CNS infection could provide valuable insight for the development of next-generation therapies for the prevention of epilepsy. Theiler’s murine encephalomyelitis virus (TMEV)-infected C57BL/6J mice have acute seizures 3–8 days postinfection (dpi) and exhibit pathologic and physiologic changes such as astrogliosis, microgliosis, neuronal loss in CA1, and increased excitatory synaptic transmission in CA3 pyramidal neurons in the hippocampus. Importantly, the mice survive the infection, clear the virus from the brain, present with cognitive impairment and anxiety-like symptoms, and develop chronic spontaneous seizures (Libbey et al., 2008; Stewart et al., 2010a; Smeal et al., 2012; Umpierre et al., 2014; Bröer et al., 2016). Thus, TMEV-infected mice recapitulate many clinical observations from patients suffering from infection-induced temporal lobe epilepsy (TLE) and provide an opportunity to study the molecular mechanisms of infection-induced epileptogenesis.

TMEV infection results in an increase in the expression of cytokines, oxidative stress markers, and infiltration of macrophages in the first week of infection, which may contribute to the initiation and propagation of acute seizures (Kirkman et al., 2010; Cusick et al., 2013; Bhuyan et al., 2015). The level of TNFα mRNA in the whole brain of TMEV-infected mice exhibiting acute seizures was increased by 128-fold at 6 dpi compared with control mice (Kirkman et al., 2010). Further, only 10% of TNFα receptor 1 knockout (TNFR1 KO) mice developed acute behavioral seizures compared with 52% of wild-type (WT) mice, which implies a significant role of TNFR1-mediated effects of TNFα in seizure development in this model (Kirkman et al., 2010).

TNFα contributes to the regulation of homeostatic synaptic scaling through the TNFR1 pathway by modulating the postsynaptic expression of α-amino-3-hydroxy-5-methyl-4-isoxazolepropionic acid receptors (AMPARs) under both physiologic and pathogenic conditions (Beattie et al., 2010). TNFα increases the expression of GluA1-containing and GluA2-lacking AMPARs on the surface of cultured hippocampal neurons and in pyramidal cells in rat hippocampal slices (Beattie et al., 2002; Stellwagen et al., 2005; Stellwagen and Malenka, 2006). Rat hippocampal slices pretreated with TNFα show significant increases in average amplitudes of miniature excitatory postsynaptic currents (mEPSCs) in CA1 pyramidal neurons, suggesting that TNFα may contribute to hyperexcitability (Stellwagen et al., 2005). The effects of TNFα on regulating excitatory synaptic strength have also been described in rodent models of spinal injury (Ferguson et al., 2008), pain (Choi et al., 2010), and glaucoma (Cueva Vargas et al., 2015).

The present experiments were performed to evaluate the role of hippocampal TNFα signaling in TMEV-induced acute seizure generation. We found a significant increase in expression of both hippocampal mRNA and protein levels of TNFα after TMEV infection that was coincident with focal and generalized seizure activity recorded by video-electroencephalography (vEEG). Further, a significant increase in the TNFR1:TNFR2 ratio in hippocampus suggests that TNFR1-mediated signaling predominates during the acute infection period. Consistent with increased TNFR1 signaling, increases in hippocampal cell-surface AMPA receptor expression were also observed during the acute period. Although treatment with XPro1595, a mutant form of human soluble TNFα (sTNFα) that acts as a dominant-negative inhibitor of sTNFα, had no effect on acute seizures, several lines of genetically modified animals deficient in either TNFα or its receptors were found to have robust changes in seizure incidence and severity after TMEV infection. In contrast to TNFR1^–/–^ mice examined previously (Kirkman et al., 2010), TNFR2^–/–^ mice developed severe acute seizures, suggesting that TNFR2-mediated signaling may be beneficial during the acute seizure period. In conclusion, the present results suggest that increases in TNFα signaling, likely through the TNFR1 pathway, contribute to hyperexcitability and the increased probability of seizures in the hippocampus after TMEV infection. Therefore, this pathway may provide a novel target for antiseizure and disease-modifying treatments after CNS infection.

## Materials and Methods

### Animals

C57BL/6J mice [WT (#006460), and breeding pairs of TNFR2^–/–^ (#002620), TNFR1^–/–^TNFR2^–/–^ (#003243), and TNFα^–/–^ (#005540)] aged 4–6 wks were purchased from The Jackson Laboratory. TNFR2^–/–^, TNFR1^–/–^TNFR2^–/–^, and TNFα^–/–^ mice were bred at our vivarium. All the KO mice were on C57BL/6J background, and deletion of the target protein was confirmed by PCR. All the experiments were conducted in male mice unless otherwise specified. After arrival, mice were allowed to acclimatize for at least 3 d before the experiment. Mice were provided food and water *ad libitum* and kept in a facility providing 12 h of light and dark cycle starting at 6:00 am. All the procedures performed were in accordance with the guidelines provided and approved by the Institutional Animal Care and Use Committee of the University of Utah.

### Method of TMEV infection and seizure monitoring

Mice are briefly anesthetized with 3% isoflurane and injected with 20 µl of either PBS or Daniels strain (DA)-TMEV solution intracortically in the right hemisphere by inserting the needle at a 90° angle to the skull. The injection region is located slightly medial to the equidistant point on the imaginary line connecting the eye and the ear. A sterilized syringe containing a plastic jacket on the needle exposing 2.5 mm of needle is used for infection to restrict the injection site to the somatosensory cortex without damaging the hippocampus. TMEV titer injected per mouse ranged from 2 × 10^4^ to 3 × 10^5^ pfu, depending on the experiment.

**Figure 1.**
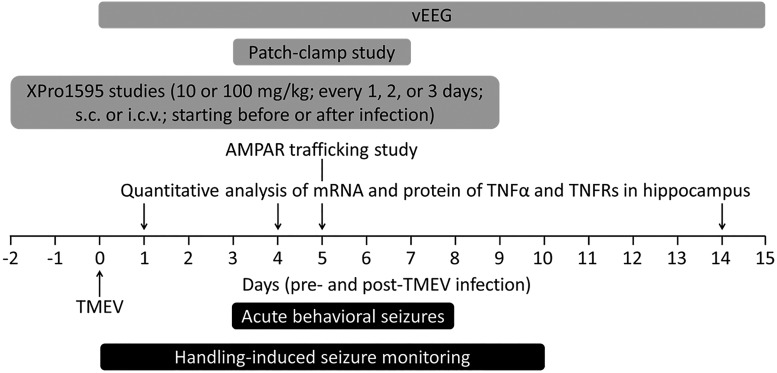
Timeline of TMEV infection in mice, acute seizure monitoring, and biochemical, molecular, electrophysiological, and pharmacological experiments reported in this article. Negative numbers on the axis indicate days before TMEV infection. vEEG was conducted for more than 1 mo continuously immediately after infection but is shown only through 15 dpi because we had to exclude several mice from the study owing to loss of the electrode assembly after 15 dpi. Various dosing paradigms of XPro1595 were tested for their effects on TMEV-induced acute seizures as described in detail in Results.

Mice were briefly agitated by shaking their cages and were monitored for behavioral seizures twice a day in the morning and afternoon, separated by a minimum of 2 h, until 10 dpi. Seizure intensity was graded using a modified Racine scale as follows: stage 1, mouth and facial movements; stage 2, head nodding; stage 3, forelimb clonus; stage 4, forelimb clonus, rearing; stage 5, forelimb clonus, rearing, and falling; and stage 6, intense running, jumping, repeated falling, and severe clonus (Racine, 1972). Seizure frequency was reported as an average number of seizures during the entire acute seizure period, whereas seizure severity/intensity was represented as an average cumulative seizure burden at each dpi during acute seizure period. Cumulative seizure burden at each dpi for a mouse was calculated by summing all of its seizure scores up to that dpi. The timeline for TMEV infection, acute seizure monitoring, and all experiments conducted is shown in Fig. 1.

### Video-electroencephalography

Twenty C57BL6/J male mice were anesthetized with the solution of ketamine and xylazine (80 mg/kg ketamine, 12 mg/kg xylazine, intraperitoneal (i.p.)) and implanted with a bipolar electrode (Plastics One) in the dentate gyrus using stereotaxic coordinates of 1.43 mm lateral (ipsilateral to the infection site), 2.46 mm posterior, and 2.35 mm ventral from the bregma. Two anchor screws were placed in the skull over the left and right frontal parietal cortex, and the third screw was anchored over the left parietal cortex. The electrode and all the screws were secured in position using glue (Loctite 454). After 2 wks of recovery, the animals were injected with 20 µl TMEV (3 × 10^5^ pfu) or PBS in the right somatosensory cortex as described above. The animals were then enrolled for vEEG studies by connecting to an EEG100C amplifier (BioPac Systems) using a tether with a rotating commutator. The experimental setup for the vEEG was custom designed to allow for continuous monitoring of EEG and corresponding video recording of each animal 24 h/d, 7 d/wk (Thomson and White, 2014). Mice had convenient access to food and water during the entire vEEG study. The EEG recording was bandpass filtered between 1 and 100 Hz at a sampling rate of 500 Hz. The EEG and video recordings were reviewed manually by an experimenter blinded to the treatment groups. Electrographic seizures were defined as rhythmic spikes or sharp-wave discharges with amplitudes at least two times higher than baseline and lasting at least 5 s. The EEG recoding associated with any seizure activity including convulsive and nonconvulsive is accompanied by postictal suppression corresponding to behavioral arrest in mice, whereas the electrographic artifacts associated with mouse behavior other than seizures are not accompanied by suppression of the basal EEG activity. By verifying the suppression of the basal EEG activity as well as observing the video recording, seizures were identified and artifacts were excluded from the analysis. For the purpose of analysis, the seizures were categorized as focal seizures if the electrographic seizures were not accompanied by tonic-clonic seizures and as generalized seizures if the electrographic seizures were accompanied by tonic-clonic seizures. The seizure severity was determined using a modified Racine scale as described above. At the end of the experiment, mice were transcardially perfused with ice-cold PBS followed by 4% paraformaldehyde, and brains were collected. The placement of the electrode was verified by staining the brain slices with 0.1% cresyl violet. However, in some cases, animals died before perfusing them, and severe hippocampal sclerosis in some TMEV-infected animals prevented definitive identification of electrode site.

### Tissue collection for mRNA and protein analysis

Because the majority of mice infected with TMEV developed handling-induced behavioral seizures, all the biochemical and molecular studies were conducted in TMEV-infected mice that had acute behavioral seizures. TMEV- and PBS-treated mice were killed at 1, 4, 5, and 14 dpi depending on the experiment. The ipsilateral and contralateral hippocampi were rapidly isolated and collected separately. All the tissue samples were flash-frozen using 2-methylbutane chilled on dry ice and stored at –80°C until further processing. Because the present vEEG experiments and prior electrophysiology experiments in the TMEV model were performed in the ipsilateral hippocampus, we conducted biochemical and molecular experiments in ipsilateral hippocampus.

### Multiplex cytokine array

The protein levels of an array of cytokines were measured in mouse ipsilateral hippocampal lysates using a multiplex kit (V-PLEX, K15048D) from Mesoscale Discovery according to the manufacturer’s instructions. Briefly, ipsilateral hippocampi were homogenized in MSD Tris lysis buffer in a 1:10 weight-by-volume ratio and centrifuged, and the supernatant was collected. Protein concentration was determined using the Bradford assay, and 250 µg protein was loaded per well to measure cytokines. Levels of analytes were determined by measuring the intensity of emitted light at 620 nm using a Sector Imager 2400.

### Gel electrophoresis and Western blot

Protein expression of TNFRs was quantified by Western blot analysis. The ipsilateral hippocampi were homogenized in 10 µl lysis buffer [25 mm Tris-HCl, 150 mm NaCl, 1 mm EDTA, 1% Igepal CA-630, 5% glycerol, protease inhibitors (cocktail tablet, Roche), and 1 mm sodium orthovanadate] per mg of tissue, and the supernatant was collected after centrifugation. Total protein concentration was measured by BCA protein assay (Pierce), and 10 µg total protein was electrophoresed using polyacrylamide gel (4–12% Bis-Tris gel, NuPAGE; Invitrogen) under denaturing conditions. The proteins were transferred to a PVDF membrane and detected by chemiluminescence (NEL105001EA; PerkinElmer) using rabbit polyclonal antibodies against TNFR1 (#ab64006, 1:25,000; Abcam), TNFR2 (#3727, 1:1000; Cell Signaling Technologies), and actin (#A2103, 1:250,000, Sigma-Aldrich) followed by horseradish peroxidase–conjugated secondary antibody (#65-6120, 1:3000; Invitrogen). Densitometric analysis of protein levels was performed using ImageJ software (NIH).

### Quantitative reverse transcription PCR

Total RNA was isolated from the hippocampi samples by Trizol/chloroform extraction and purified to remove genomic DNA contamination by DNase treatment followed by a spin column–based method (RNeasy Mini Kit; Qiagen) according to the manufacturer’s instructions. Quality and quantity of RNA were validated by spectrophotometry and acrylamide gel electrophoresis, which showed intense discrete ribosomal RNA bands devoid of genomic DNA. cDNA was synthesized from RNA using random primers (SuperScript VILO Master Mix; Invitrogen) and amplified by qPCR (LightCycler480; Roche) using 1 µg cDNA with Luminaris Color HiGreen qPCR Master Mix (Thermo Fisher Scientific) and 0.3 µm primers for TNFR1 (forward, 5′-AGAGAAAGTGAGTGCGTCCC-3′; reverse, 5′-AGCCTTCTCCTCTTTGAC-AGG-3′), TNFR2 (F, 5′-AGCTGCAGTTCTTCCTGTACC-3′; R, 5′-GATGCTACA-GATGCGGTGGG-3′), TNFα (F, 5′-CTGAACTTCGGGGTGATCGG-3′; R, 5′-GGC-TTGTCACTCGAATTTTGAGA-3′, β-actin (F, 5′-AGATCAAGATCATTGCTCC-TCC-3′; R, 5′-ACGCAGCTCAGTAACAGTCC-3′), and GAPDH (F, 5′-AGCTAC-TCGCGGCTTTACG-3′; R, 5′-GGCCAAATCCGTTCACACC-3′). LC480 software used a second derivative formula to calculate threshold cycle (C_T_). Primer efficiency for each analyte was determined using four dilutions of cDNA, and the primer efficiency was used to calculate ΔC_T_ and ΔΔC_T_ relative to the reference gene as well as PBS-treated control group. Primer specificity was confirmed by melting curves followed by agarose gel electrophoresis for the expected-size product.

### Treatment of TMEV-infected mice with XPro1595

TMEV-infected mice were treated with 10 or 100 mg/kg XPro1595 subcutaneously. Vehicle contained 150 mm NaCl, 10 mm l-histidine, and 0.01% w/v Tween 20 in deionized water (pH 6.51). For intracerebroventricular (i.c.v.) administration of XPro1595 subcutaneously (s.c.), the guide cannula was surgically implanted into the left lateral ventricle using stereotaxic coordinates of –1.1 mm lateral, –0.5 mm posterior, and –3.0 mm ventral from the bregma as described in detail previously (DeVos and Miller, 2013). For implantation, mice were anesthetized by 10 mL/kg i.p. injection of a mixture of ketamine and xylazine solution in sterile PBS (final concentration: ketamine ,10.4 mg/ml; xylazine, 1.6 mg/ml), and the skull was exposed in a sterile surgical environment. Guide cannulas (C315GS-5/SP, 3 mm below pedestal; PlasticsOne) were inserted into the left lateral ventricle using a sharp beveled end of the cannula and glued to the skull. A dummy cannula (C315DCS-5/SPC; PlasticsOne) was placed in the guide cannula to prevent exposure of the ventricle to the outside environment. Mice were allowed to recover from surgery for 12–15 d before the experiment. Mice were immobilized during drug infusion by anesthetizing them using isoflurane (VetEquip isoflurane vaporizer), and the dummy cannula was removed just before the drug infusion in a laminar flow hood under aseptic conditions. An internal cannula (C315IS-5/SPC; PlasticsOne) connected to a 10-µl Hamilton syringe via tubing was inserted into the guide cannula to infuse either XPro1585 or the vehicle solution. An infusion pump (PHD 2000 programmable; Harvard Apparatus) was used to infuse either 5 µl of 40 mg/ml XPro1595 at 1 µl/min (0 and 2 dpi) or 2.5 µl of 80 mg/ml XPro1595 at 0.5 µl/min (4 and 6 dpi). The internal cannula was kept in place for ∼1 min after infusion and then slowly removed to avoid leakage. The dummy cannula was secured into the guide cannula immediately. At the end of the experiment, cannula placement was confirmed for each animal.

### Cell-surface biotinylation assay

Horizontal brain slices (350 µm) from control and TMEV-infected mice with seizures were prepared by vibratome at 5 dpi in ice-cold sucrose solution (concentrations in mm: 200 sucrose, 3 KCl, 26 NaHCO_3_, 1.4 NaH_2_PO_4_, 10 glucose, and 3 MgSO_4_ and 1 CaCl_2_ added before use; 4°C) and collected in artificial CSF (aCSF, concentrations in mm: 126 NaCl, 3 KCl, 26 NaHCO_3_, 1.4 NaH_2_PO_4_, 10 glucose, and 2 MgSO_4_ and 2 CaCl_2_ added before use; pH, 7.33–7.35; osmolality, 297–303 mOsm/kg) at room temperature. Only brain slices ipsilateral to the injection were used for further processing. The slices were incubated in aCSF (31°C) for 45–50 min to recover from the surface damage inflicted during slicing. All the remaining steps were conducted at 4°C. The slices were washed with aCSF to remove dead surface cells and debris and incubated in 1 mg/ml solution of sulfo-NHS-SS-biotin (Pierce) for 30 min to biotinylate cell-surface proteins. The excess biotin solution was washed off using aCSF, quenched by incubating the slices in 100 mm glycine solution, and again washed with aCSF. The hippocampal regions were dissected out quickly, collected in 250 µl lysis buffer (recipe same as described in Western blot procedure), homogenized, and centrifuged (14.000 × *g*, 15 min, 4°C), and the supernatant was collected. All the solutions used for processing brains and brain slices were continuously oxygenated with a mixture of 95% oxygen and 5% carbon dioxide.

Total protein (biotinylated surface proteins and nonbiotinylated intracellular proteins) concentrations in the supernatant were measured by a BCA protein assay (Pierce). To isolate biotinylated surface proteins (SP) from the total proteins (TP), 50 µg TP was incubated with 25 µl streptavidin beads (NeutrAvidin; Thermo Fisher Scientific) overnight at 4°C on a rotator. The appropriate ratio of beads to TP for each protein of interest was empirically measured by incubating a constant volume of beads with a range of TP to isolate the corresponding SP, as described in detail previously (Gabriel et al., 2014). We chose a ratio of beads to TP of 1:2, which was found to isolate SP in a linear range. The mixture of beads and TP was centrifuged, and the beads were washed with lysis buffer. SP were eluted by incubating the beads in 20 µl of 2× SDS-PAGE reducing sample buffer containing 50 mM dithiothreitol in the final mixture (4× SDS-PAGE sample buffer: 106 mm Tris-HCl, 141 mm Tris-base, 0.51 mm EDTA, 2% SDS, and 10% glycerol) for 30 min with continuous gentle mixing at room temperature. The supernatant containing SP was collected after centrifugation (17,000 × *g*, 2 min). The expression of GluA1 and GluA2 subunits of AMPARs were measured in both SP and TP fractions by SDS-PAGE and Western blot (anti-GluA1 mAb #MAB2263 and anti-GluA2 mAb #MAB397; EMD Millipore) as described above. The entire volume of supernatant containing SP (isolated from 50 µg TP) and 10 µg TP were electrophoresed in the same gel.

### Patch-clamp electrophysiology

Mice were killed at 3–7 dpi, their brains were removed, and 350-µM horizontal brain slices were cut in ice-cold sucrose solution and incubated in aCSF solution for 1 h at room temperature. Miniature EPSCs were recorded from the dentate granule cells (DGCs) by whole-cell patch-clamp in aCSF solution containing 1 µm tetrodotoxin as described previously (Smeal et al., 2012). The internal recording solution contained (in mm): 129 potassium gluconate, 6 CsCl, 10 Hepes, 1 EGTA, 0.5 CaCl_2,_ 10 glucose, 2 ATP, 0.5 GTP, 5 QX314, 1 NaCl, and 5 tetraethylammonium chloride. The equilibrium potential for ionotropic glutamate receptors (iGluR E_Na+/K+_) was 4.66 mV at room temperature, and mEPSCs were recorded by clamping the cell at –70 mV. Properties of the miniature currents analyzed included amplitude, interevent interval, frequency, rise time, and decay time (Smeal et al., 2012).

### Statistics

Datasets involving continuous variables are represented by the average and SEM, and the datasets with ordinal variables by frequency distribution. Experimental design involving two groups with one continuous dependent variable was analyzed by unpaired two-tailed *t* test, whereas designs involving more than two groups with two categorical independent variables and one continuous dependent variable was analyzed by two-way ANOVA. Multiple comparisons were performed by Bonferroni posttest. Average cumulative seizure burden, which was calculated from a ranked dataset, was analyzed by Scheirer–Ray–Hare test, which is an extension of the Kruskal–Wallis test for two randomized factorial designs (Scheirer et al., 1976). Two groups with binomial outcome were analyzed by Fisher’s exact test. Survival (% seizure free) curves and cumulative distributions were analyzed by log-rank test and Kolmogorov–Smirnov test, respectively. Densitometry of immunoblot images and the analysis of mEPSCs were conducted with ImageJ and Mini Analysis Program (version 6.0.7; Synaptosoft), respectively. Statistical calculations were conducted using GraphPad Prism 5 and Microsoft Excel.

## Results

### Focal seizures occur in the hippocampus during the acute infection period after TMEV inoculation

**Figure 2.**
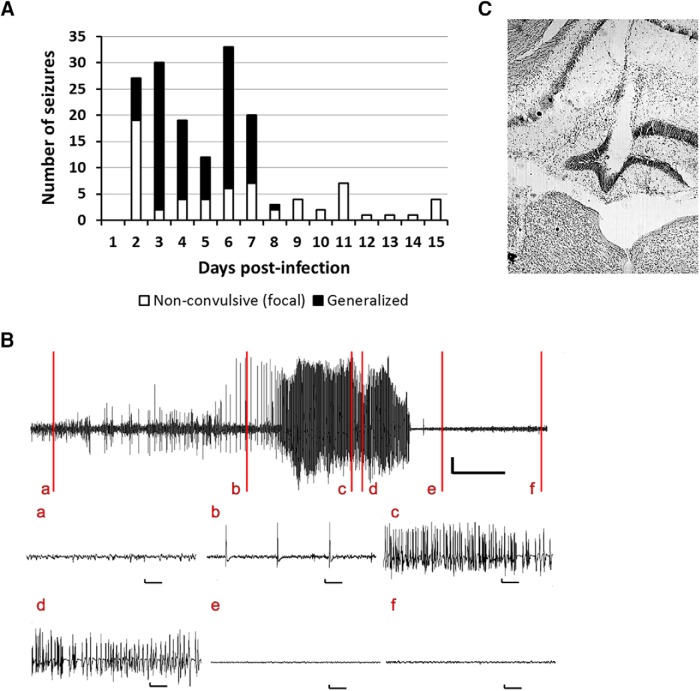
Mice implanted with an electrode in the dentate gyrus for 24 h/7 d/wk vEEG show focal as well as generalized seizures after TMEV infection. (A) Total number of acute seizures each day after TMEV infection from all mice that developed acute seizures. Of 16 mice implanted with an electrode in the dentate gyrus and infected with 3 × 10^5^ pfu TMEV, 11 developed acute seizures. The mice had nonconvulsive focal seizures (white bar) as well as spontaneous generalized seizures (black bar) during the first week after TMEV infection; however, nonconvulsive focal seizures continued to occur in some mice after acute behavioral seizures subsided. Although the vEEG was conducted for more than 1 mo postinfection, the seizure data are shown until 15 dpi, as several mice were discontinued from the study after 15 dpi owing to loss of electrode assembly. (B) Representative trace of vEEG recording from a mouse at 5 d after TMEV infection. The vertical lines on the upper vEEG trace indicate baseline EEG during normal mouse behavior (a); nonconvulsive focal electrographic seizure (b); stage 3 behavioral seizure (c); stage 4 behavioral seizure (d); stage 4 behavioral seizure without electrographic activity (e); and normal mouse behavior and the electrographic activity returning to baseline (f). All vertical scale bars represent 500 µV, and the horizontal scale bars represent 30 s in the upper vEEG trace and 1 s in each of the expanded traces below. (C) Location of the electrode in the molecular layer of the dentate gyrus (near the center of the image). The coronal brain slice shown here, stained with 0.1% cresyl violet, was obtained from a TMEV-infected mouse that had seizures.

Previous vEEG experiments using cortical surface electrodes have demonstrated that TMEV-infected mice have limbic seizures that secondarily generalize during the acute infection period (Stewart et al., 2010a; Bröer et al., 2016). Because TMEV infection results in significant cell loss in the CA1 region and increased excitability in the CA3 region of the hippocampus, a brain region often associated with seizure initiation in TLE, we hypothesized that focal seizures occurring in the hippocampus might not have been observable with cortical electrodes as used in a prior study (Stewart et al., 2010a). To test the hypothesis that focal seizures occur in the hippocampus, a depth electrode was implanted in the dentate gyrus (DG) region of the dorsal hippocampus of mice 2 wks before TMEV infection (*n* = 16). Although no seizures were observed in the mice injected intracortically with PBS (*n* = 4, data not shown), 11 of the 16 mice (68%) injected with TMEV exhibited both focal and secondarily generalized seizures during the recording period (Fig. 2*A*). Consistent with previous results, generalized tonic-clonic seizures occurred during the first several days after infection. The behavioral aspects of many generalized seizures were preceded by electrographic seizure activity in the hippocampus, and the electrographic seizures stopped earlier than behavioral seizures (Fig. 2*B*; Video 1). In addition, many of the seizures recorded in the DG did not secondarily generalize and were thus classified as focal seizures (Fig. 2*A*). Interestingly, although the generalized seizures largely remitted after day 8, focal seizures continued to occur in several of the mice after that time point. No overt behavioral correlates accompanied these focal seizures. This suggests that significant epileptiform activity and hyperexcitability occurred in the hippocampus as a consequence of TMEV infection. The placement of the electrode in the dentate gyrus was verified by immunohistochemical staining with 0.1% cresyl violet after completion of the vEEG study. Fig. 2*C* shows the placement of the electrode in the molecular layer of the DG in a TMEV-infected mouse that had seizures. Because the EEG recordings were not acquired from multiple sites in this study, it is not possible to determine whether the seizures originated in the hippocampus per se (Toyoda et al., 2013). Further studies are required to map the brain circuit involved in seizure initiation and propagation. In addition, owing to the loss of some animals during the recording period and severe hippocampal sclerosis in some of the TMEV-infected mice, it was not always possible to definitively confirm electrode placement in some animals. Nevertheless, the initial occurrence of seizures in the hippocampus before secondary generalization suggests that there is considerable hyperexcitability within the hippocampus, consistent with prior observations with c-Fos immunocytochemistry demonstrating activation of this immediate early gene in the hippocampus within 2 h after TMEV-induced acute seizures, and consistent with increases in excitatory synaptic transmission in the hippocampus (Smeal et al., 2012).

Video 1. Example of a vEEG recording from a mouse implanted with an electrode in the dentate gyrus. The vEEG recording was obtained at 5 d after TMEV infection. It is noted that the behavioral aspects of the generalized tonic-clonic seizure in this mouse are preceded by an electrographic seizure activity in the hippocampus, and the electrographic seizure stops earlier than the behavioral seizure. 10.1523/ENEURO.0105-17.2017.video.1

### mEPSCs recorded in DGCs are not affected by TMEV infection

**Figure 3.**
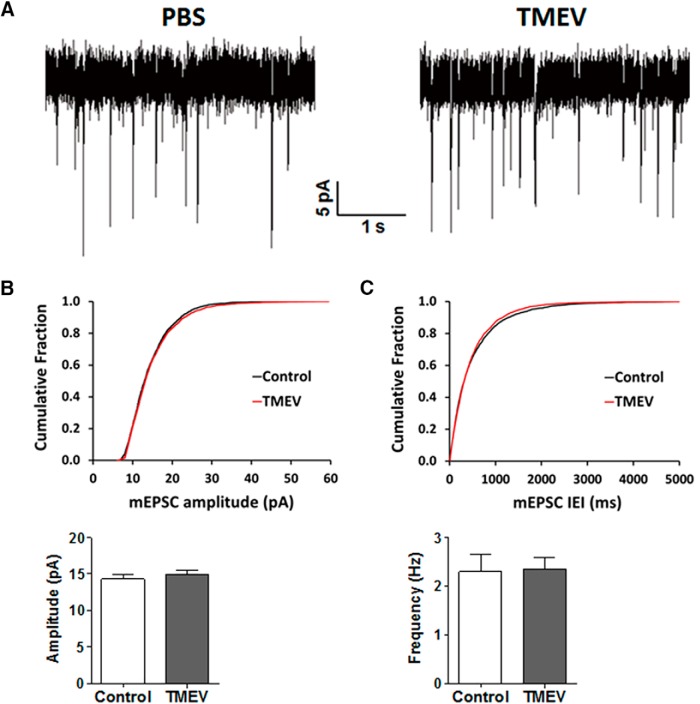
No difference in the properties of mEPSCs of DGCs recorded in brain slices obtained from PBS-injected (control) and TMEV-infected mice during the acute seizure period 3–7 dpi. (A) Representative traces of mEPSCs measured in the DGCs from the control group and TMEV-infected mice at 5 dpi. (B) Cumulative fraction distribution of the amplitude of mEPSCs shows no difference between control and TMEV groups. Average amplitudes of mEPSCs are plotted in the lower panel (control, *n* = 8; TMEV, *n* = 10). (C) Cumulative fraction distribution of the interevent interval (IEI) of mEPSCs shows no difference between treatment groups. The lower panel shows the average frequency of mEPSCs. Statistics: Kolmogorov–Smirnov test (cumulative fraction), unpaired *t* test (average amplitude and frequency).

TMEV exhibits a strong tropism for limbic brain regions, and viral particles can be detected abundantly in CA1 and CA2 regions of hippocampus in the first week of infection (Buenz et al., 2009; Kirkman et al., 2010; Stewart et al., 2010b). The extent of neuronal damage induced by TMEV varies across different regions of hippocampus, ranging from severe to moderate to negligible in the pyramidal cells of CA1, CA3, and the granule cells of the DG, respectively (Stewart et al., 2010a). Abundant c-Fos immunoreactivity, an indirect marker for neuronal activation, was found in the CA3 region and the DG of hippocampus within 2 h after TMEV-induced acute seizures (Smeal et al., 2012). In addition, patch-clamp studies in acute brain slices found an increase in the amplitude and frequency of spontaneous and miniature EPSCs in CA3 pyramidal neurons during the period of 3–7 days after TMEV infection (Smeal et al., 2012). Because we observed the appearance of seizures in the hippocampus before secondary generalization, we performed whole-cell patch-clamp recordings in DGCs to determine whether there was an increase, as in CA3, in excitatory synaptic transmission during the acute infection period when seizures occur. Miniature EPSCs in DGCs in hippocampal brain slices were obtained during the acute seizure period. Representative traces of mEPSCs from control and TMEV-infected mice are shown in Fig. 3*A*. A total of 11 DGCs from 8 mice in the control group and 15 DGCs from 10 TMEV-infected mice with acute seizures were included in the analysis. The cumulative fraction analysis of mEPSC amplitude as well as interevent interval found no significant differences between treatment groups (*p* > 0.05, KS test; Fig. 3*B*, *C*). The average (± SEM) mEPSC amplitudes were 14.3 ± 0.66 and 14.9 ± 0.59 pA in the control and TMEV mice, respectively (*p* = 0.5), and the frequencies were 2.30 ± 0.36 and 2.35 ± 0.23 Hz (*p* = 0.9). These data suggest that, unlike CA3 pyramidal neurons, DGCs do not have an increase in excitatory synaptic transmission during the acute infection period.

### Increased expression of TNFα and other cytokines in the hippocampus after TMEV infection

**Figure 4.**
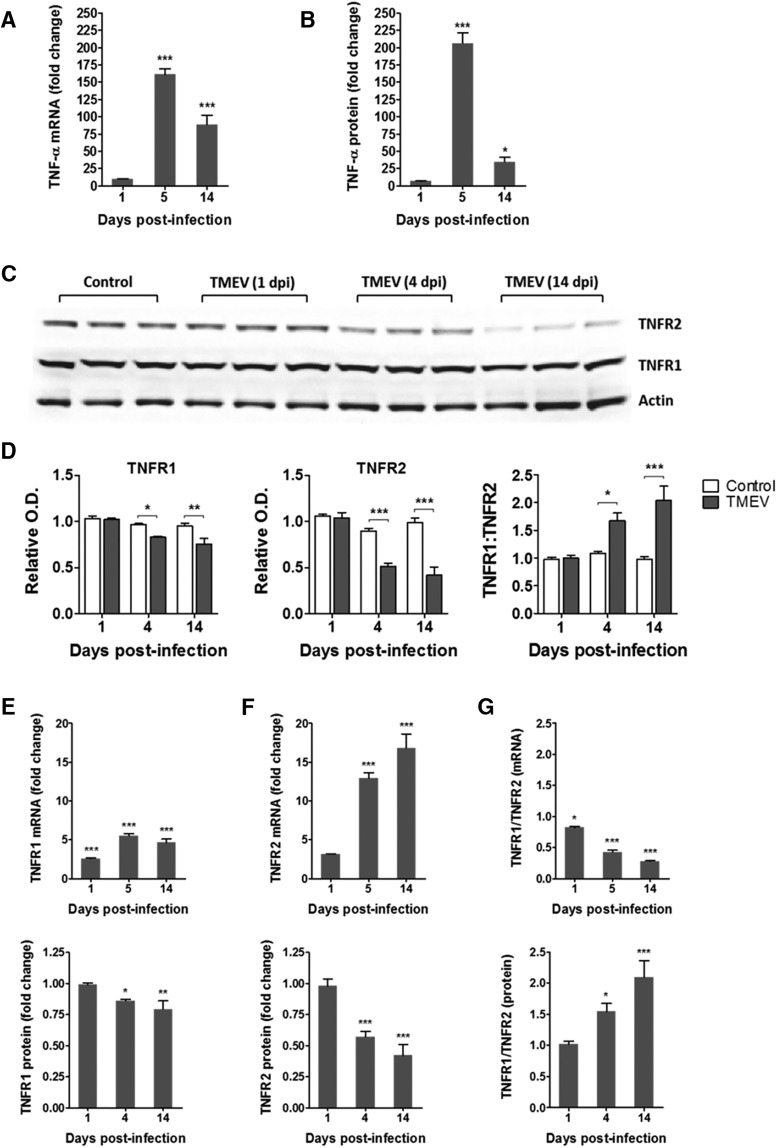
Increase in the levels of TNFα and in a ratio of the protein expression of TNFR1:TNFR2 in the hippocampus of TMEV-infected mice during acute seizure activity period. (A) mRNA levels of TNFα, as measured by RT-qPCR, are significantly increased in TMEV-infected mice at 5 and 14 dpi by 161- and 88-fold, respectively, compared with PBS-infected control mice (*n* = 4 for TMEV and control). (B) 206- and 35-fold increase in the protein expression levels of TNFα in TMEV-infected mice at 5 and 14 dpi compared with the PBS-injected control mice [control, *n* = 5; TMEV, *n* = 8 (1 dpi), 6 (5 dpi), and 5 (14 dpi)]. (C) Representative immunoblot shows the protein expression of TNFR1, TNFR2, and actin in the hippocampus from PBS- and TMEV-infected mice (*n* = 3). (D) Densitometric analysis of the immunoblots shows the expressions of TNFR1 and TNFR2 normalized to the expression levels of actin [control, *n* = 5; TMEV, *n* = 5 (1 dpi), 6 (4 and 14 dpi)] O.D., optical density. Relative expression levels of TNFR1 and TNFR2 (TNFR1:TNFR2) are significantly increased by 1.54- and 2.1-fold at 4 and 14 dpi, respectively, in TMEV-infected mice compared with control mice. (E) mRNA levels of TNFR1 and TNFR2 in the hippocampus of TMEV-infected mice before (1 dpi), during (5 dpi), and after (14 dpi) acute seizures. The ratio of TNFR1 to TNFR2 mRNA is significantly reduced during the acute infection period. Data are shown as mean ± SEM. Statistics: two-way ANOVA followed by Bonferroni posttest; *, *p* < 0.05; **, *p* < 0.01; ***, *p* < 0.001.

Previous studies have demonstrated that TNFα mRNA levels increase dramatically by 128-fold in whole brain homogenates of TMEV-infected mice exhibiting acute behavioral seizures at 6 dpi compared with noninfected control mice (Kirkman et al., 2010). However, given the occurrence of focal seizures in the hippocampus, it is important to determine the role of hippocampal TNFα in TMEV-induced seizures. Therefore, we measured the expression levels of TNFα mRNA and protein in the hippocampus of TMEV-infected mice and PBS-injected control mice. TMEV-infected mice exhibit acute behavioral and electrographic seizures at 3–8 dpi. Therefore, we measured the levels of TNFα at 1, 5, and 14 dpi to compare the levels before, during, and after the acute seizure period. Only handling-induced behavioral seizures were evaluated in these and the following studies, as the presence of depth electrodes could influence the cytokine response. The mRNA level of TNFα in TMEV-infected mice was not significantly different at 1 dpi compared with control mice (*n* = 4; Fig. 4*A*). However, it dramatically increased, by 161-fold (*n* = 4, *p* < 0.001), at 5 dpi during the peak of the acute seizure activity, and it was still 88-fold higher at 14 dpi (*n* = 4, *p* < 0.001; Fig. 4*A*). Similarly, immunoassay studies found no significant increase in the protein expression of TNFα in the hippocampus of TMEV-infected mice compared with control mice at 1 dpi, but TNFα was elevated 206-fold (*p* < 0.001) and 35-fold (*p* < 0.05) at 5 and 14 dpi, respectively [Fig. 4*B*, Table 1; control, *n* = 5 (all time points); TMEV, *n* = 8 (1 dpi), 6 (5 dpi), and 5 (14 dpi)]. The absolute levels of TNFα were measured as follows: 4.89 ± 1.19 pg/ml (control) and 33.80 ± 3.09 pg/ml (TMEV) at 1 dpi; 0.54 ± 0.22 pg/ml (control) and 110.50 ± 7.96 pg/ml (TMEV) at 5 dpi; and 1.41 ± 0.65 pg/ml (control) and 48.92 ± 10.02 pg/ml (TMEV) at 14 dpi. Additionally, protein levels of many other cytokines were also increased in the hippocampus in TMEV-infected mice with seizures at 5 dpi (Table 1), notably interferon-γ (IFNγ) which was 21,734-fold elevated compared with control mice. In addition, the anti-inflammatory cytokine interleukin-10 (IL-10) was increased by 47-fold, which suggests that negative immune feedback occurs during acute TMEV infection to control excessive inflammation.

**Table 1.** Significant increase in the protein levels of various inflammatory mediators in the hippocampus of TMEV-infected mice during acute seizure activity period. Statistics: Two-way ANOVA, Bonferroni posttest, ^#^p<0.05, ^*^p<0.01, ^†^p<0.001; SEM, standard error of the mean. (TNFα, tumor necrosis factor-α; IFNγ, interferon-γ; IL, interleukin; CXCL1, C-X-C motif chemokine ligand 1)

	Fold change (relative to PBS-injected control mice)					
	1 dpi (*n* = 8)		5 dpi (*n* = 6)		14 dpi (*n* = 5)	
Cytokine	Average	SEM	Average	SEM	Average	SEM
TNFα	6.9	0.6	206.2^†^	14.9	34.8^#^	7.1
IFNγ	4.5	0.8	21,734.4^†^	3123.2	99.1	21.6
IL-1β	6.9	1.1	58.2^†^	5.4	46.6	13.5
IL-10	2.3	0.3	47.2^†^	6.0	9.6	1.9
IL-12p70	2.5	0.3	3.0^#^	0.5	6.0^†^	1.0
IL-4	1.0	0.2	0.8	0.1	1.3	0.3
IL-2	2.4	0.3	14.4^†^	4.2	4.9	0.7
IL-5	2.0	0.1	10.8*	2.5	2.6	1.2
CXCL1	4.6	0.3	30.0^†^	4.3	2.4	0.3

Statistics: two-way ANOVA, Bonferroni posttest, ^†^*p*<0.001; ^#^*p*<0.05; ^*^p<0.01. CXCL1, C-X-C motif chemokine ligand 1.

### Increased protein expression ratio of TNFR1:TNFR2 in hippocampus during acute seizures

The TNFα receptors TNFR1 and TNFR2 mediate contrasting effects of TNFα in various disease models (Fischer et al., 2015), and differential changes in the protein levels of TNFR1 and TNFR2 have been shown in the hippocampus in a rat model of limbic epilepsy (Weinberg et al., 2013). Therefore, we measured the expression of TNFR1 and TNFR2 in hippocampus after TMEV infection by Western blot. Fig. 4*C* shows a representative Western blot image for TNFR1, TNFR2, and actin (gel loading control) from TMEV-infected mice at 1, 4, and 14 dpi along with PBS-treated control mice. Optical density (OD) analysis revealed a slight but significant reduction in the expression of TNFR1 in TMEV-infected mice at 4 dpi (0.97 ± 0.012 vs. 0.83 ± 0.01, *n* = 5–6, *p* < 0.05) and 14 dpi (0.95 ± 0.03 vs. 0.75 ± 0.064, *n* = 5–6, *p* < 0.01) after normalizing the data to actin levels (Fig. 4*D*). However, the expression of TNFR2 protein levels were dramatically reduced at 4 dpi (0.90 ± 0.029 vs. 0.51 ± 0.038, *n* = 5–6, *p* < 0.001) and 14 dpi (0.99 ± 0.049 vs. 0.42 ± 0.087, *n* = 5–6, *p* < 0.001; Fig. 4*D*). The ratio of TNFR1:TNFR2, indicating the relative expressions of the two TNFRs in the hippocampus during the acute infection period, was significantly elevated (1.08 ± 0.033 vs. 1.67 ± 0139, *p* < 0.05) at 4 dpi and was further increased at 14 dpi (0.97 ± 0.057 vs. 2.04 ± 0.255, *p* < 0.001; Fig. 4*D*) which suggests that TNFα may mediate its downstream effects predominantly through TNFR1 in the hippocampus during the acute TMEV infection period.

We also measured the mRNA levels of both TNFRs using qPCR (Fig. 4*E*). In contrast to TNFα, we found opposite changes in the levels of both TNFR1 and TNFR2 for mRNA and protein expression. The expression of TNFR1 mRNA was elevated by 2.59-, 5.5-, and 4.65-fold at 1, 5, and 14 dpi, respectively (*n* = 4). The expression of TNFR2 mRNA was increased by 12.94- and 16.8-fold at 5 and 14 dpi, respectively (*n* = 4). These changes result in a progressive decrease in the relative mRNA expression ratios of TNFR1:TNFR2 in the hippocampus during acute TMEV infection (Fig. 4*E*), which is in contrast to the changes in the protein expression ratios of TNFR1:TNFR2 (Fig. 4*D*). This suggests that TNFR expression undergoes posttranslational regulation during the acute infection period.

### Lack of seizure control after peripheral administration of XPro1595 on TMEV-induced acute seizures

There is a significant increase in whole brain and hippocampal TNFα mRNA, an increase in protein expression of TNFα, and an increase in the ratio of TNFR1:TNFR2 expression in the hippocampus as a consequence of TMEV infection. In addition, prior work has demonstrated that seizure incidence is dramatically reduced in TNFR1^–/–^ mice (Kirkman et al., 2010). Therefore, we hypothesized that pharmacological inhibition of TNFR1 could decrease the incidence and severity of TMEV-induced acute seizures. TNFα is expressed as a homotrimeric transmembrane protein (tmTNFα), and the extracellular portion of tmTNFα can be cleaved by TNFα converting enzyme (TACE) to form sTNFα. Both tmTNFα and sTNFα are active in their trimeric composition and mediate a variety of cellular activities via TNFRs (McCoy and Tansey, 2008). sTNFα predominantly mediates its effects via TNFR1, whereas TNFR2 is fully activated only by tmTNFα (Grell et al., 1995, 1998). Although anti-TNFα antibodies are approved for the treatment of peripheral inflammatory conditions, they do not cross the blood–brain barrier (BBB; Tracey et al., 2008). Therefore, we selected the investigational BBB-permeant compound, XPro1595, to test the hypothesis that TNFα signaling through TNFR1 contributes to seizure generation. XPro1595 is a mutant form of human sTNFα and acts as a dominant-negative selective inhibitor of sTNFα (Steed et al., 2003). It dose-dependently exchanges with endogenous monomers of sTNFα to form an inactive heterotrimer that does not bind to TNFRs and lacks intrinsic bioactivity (Steed et al., 2003). Because sTNFα predominantly mediates its effects via TNFR1, XPro1595 indirectly inhibits TNFR1 functions. XPro1595 has been shown to provide beneficial effects in animal models of several peripheral and CNS inflammatory conditions, including experimental autoimmune encephalomyelitis, Parkinson’s disease, spinal cord injury, and focal cerebral ischemia, by selectively inhibiting sTNFα-TNFR1 signaling and sparing beneficial functions of TNFR2 (McCoy et al., 2006; Zalevsky et al., 2007; Brambilla et al., 2011; Clausen et al., 2014; Novrup et al., 2014). Because the dosing regimen of 10 mg/kg s.c. XPro1595 administered every third day was effective in models of other CNS diseases (Brambilla et al., 2011; Barnum et al., 2014; Clausen et al., 2014), we tested whether peripheral treatment of TMEV-infected mice with XPro1595 could prevent the development of handling-induced acute seizures. Handling sessions were captured by video monitoring, and experimenters scoring seizures were blinded to the treatment groups, as described in Materials and Methods. Administration of 10 mg/kg XPro1595 s.c. at 1, 4, and 7 dpi did not decrease average number of seizures per acute seizure period (3–8 dpi; vehicle, 5.27 ± 0.37; XPro1595, 5.63 ± 0.45; *n* = 30, *p* = 0.5313) or have any effect on average cumulative seizure burden at any day during 3–8 dpi compared with vehicle-treated mice. To determine whether the lack of efficacy on seizure incidence and severity was due to an inappropriate dosing regimen, we increased the dosing frequency of XPro1595 (10 mg/kg) to every day, starting from 2 h postinfection through 9 dpi. This dosing regimen was also ineffective in decreasing either average seizure frequency per acute seizure period (vehicle, 5.13 ± 0.71; XPro1595, 5.13 ± 0.77; *n* = 15, *p* = 1.00) or severity at each day during 3–8 dpi. Finally, we increased the dose to 100 mg/kg per day and started treatment 2 d before infection through 7 dpi. Despite a 10-fold increase in the dose and initiating the treatment before infection, both XPro1595 and vehicle-treated groups had similar average numbers of seizures per acute seizure period (vehicle, 6.63 ± 1.24; XPro1595, 6.25 ± 0.77; *n* = 8, *p* = 0.801) and average cumulative seizure burdens at each day during 3–8 dpi. Thus, systemic administration of high doses of XPro1595 was not effective in controlling seizures induced by TMEV infection.

### Lack of seizure control after CNS administration of XPro1595 on TMEV-induced acute seizures

**Figure 5.**
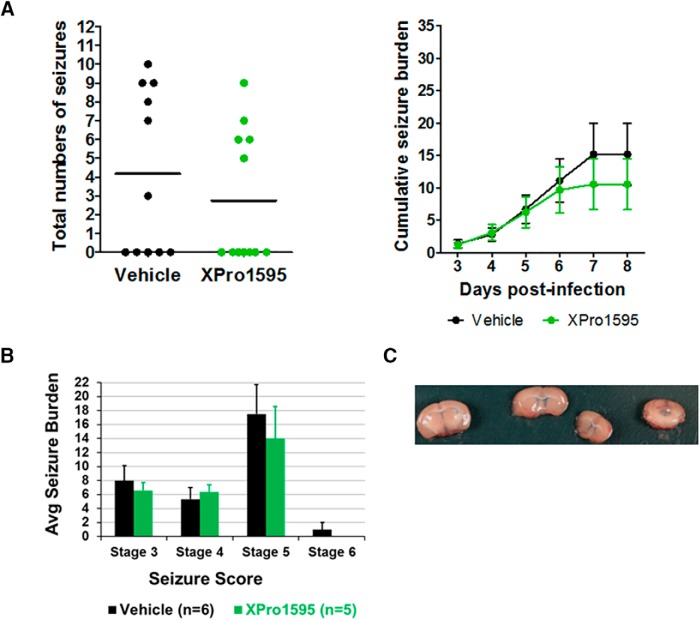
CNS administration of XPro1595 does not affect TMEV-induced acute seizure frequency and intensity. (A) Slow infusion of XPro1595 (10 mg/kg) at 0, 2, 4, and 6 dpi into the left lateral ventricle using a surgically implanted guide cannula did not reduce average seizure frequency or severity (*n* = 12, XPro1595; *n* = 11, vehicle). Each circle represents an individual mouse, and the horizontal line shows average number of seizures per group per acute seizure period (3–8 dpi). (B) Average seizure burden corresponding to each stage of modified Racine scale for generalized tonic-clonic seizures shows no difference between vehicle- and XPro1595-treated TMEV-infected mice. Only those mice that had acute seizures are included in this analysis. (C) The surgical placement of the guide cannula into the left lateral ventricle was confirmed by i.c.v. infusion of 0.1% Evans blue dye at 9 dpi in all TMEV-infected mice treated with either XPro1595 or vehicle. The panel shows an example of a diffusion of the dye into the ventricular system.

Despite numerous studies demonstrating central activity of XPro1595 in animal models of neurologic disorders, a recent study found that systemic injection of XPro1595 was insufficient to provide therapeutic efficacy in a mouse model of spinal cord injury (SCI), whereas central administration of XPro1595 provided neuroprotection and ameliorated motor dysfunction in this model (Novrup et al., 2014). The concentration of XPro1595 in the CSF (1–6 ng/ml) has been found to be 1000-fold reduced compared with that in the plasma (1–8 µg/ml) from rats after 2–3 d of treatment with XPro1595 (10 mg/kg s.c.; Barnum et al., 2014). Furthermore, as in the model of SCI, in which acute inflammatory response occurs rapidly in the CNS, the protein levels of TNFα and other inflammatory cytokines increase rapidly in the hippocampus after TMEV infection. Thus, we hypothesized that XPro1595 did not achieve a sufficient concentration in the brain to prevent signaling through the TNFR1 system after subcutaneous treatment in TMEV-infected mice. Therefore, we tested the effectiveness of CNS administration of XPro1595 by implanting the mice with guide cannulas into the left lateral ventricle. Infusions of XPro1595 (10 mg/kg starting at 5–6 h after infection) or vehicle were repeated every other day for a total of four infusions. As was observed after systemic injections, central administration of XPro1595 had no effect on either average number of seizures per acute seizure period (vehicle, 4.18 ± 1.32; XPro1595, 2.75 ± 1.02; *n* = 11-12, *p* = 0.3952) or average cumulative seizure burden at any day during 3–8 dpi (Fig. 5*A*, *B*) compared with TMEV-treated mice receiving vehicle infusions. The placement of the guide cannula into the ventricle was confirmed in all the mice enrolled for this study by infusing 0.1% Evans blue dye at 9 dpi (Fig. 5*C*). Therefore, regardless of administration route, dosing regimen, or dose, XPro1595 was ineffective in preventing seizures after TMEV infections.

### Differential susceptibility of TNFRs KO mice to develop TMEV-induced acute seizures

**Figure 6.**
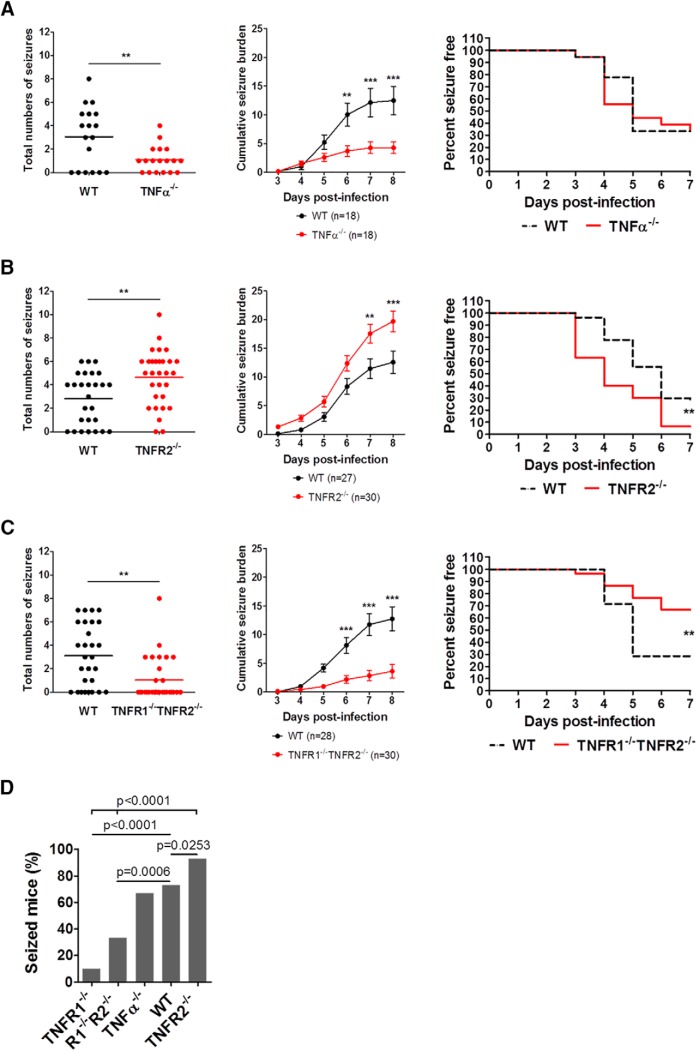
TMEV-induced acute behavioral seizure susceptibility in WT, TNFα^–/–^, TNFR2^–/–^, and TNFR1^–/–^TNFR2^–/–^ mice. (A) TNFα^–/–^ mice have a significant reduction in the seizure frequency (upper panel), plotted as a total number of seizures per mouse, and in the seizure severity (lower panel) as measured by an average cumulative seizure burden during acute seizure period (3–8 dpi) compared with WT mice. Each circle represents data from an individual mouse, and the horizontal line shows an average number of seizures per group. (B) TNFR2^–/–^ mice show an increase in average seizure frequency as well as severity. Latency to exhibit the first TMEV-induced acute seizure is reduced in TNFR2^–/–^ mice compared with WT mice. (C) Reduced average seizure frequency and severity in TNFR1^–/–^TNFR2^–/–^ mice. (D) Percentage of total infected mice that show acute behavioral seizures during 3–8 dpi. The data for TNFR1^–/–^ mice are from Kirkman et al. (2010) and are included here for comparison. Statistics: unpaired *t* test (frequency), Scheirer–Ray–Hare test (severity), Fisher’s exact test (% seized mice), and long-rank test (% seizure free); **, *p* < 0.01; ***, *p* < 0.001.

Previous work has demonstrated that only 10% of TNFR1^–/–^ mice (*n* = 2/20) infected with 2 × 10^4^ pfu of TMEV developed acute behavioral seizures, suggesting that TNFR1-mediated effects could be involved in seizure generation in this model (Kirkman et al., 2010). Because XPro1595 did not block acute seizures, we used genetically modified animals to further test the hypothesis that TNFα signaling contributes to hyperexcitability after infection. Seizure incidence and severity in TNFα^–/–^, TNFR2^–/–^, and TNFR1^–/–^TNFR2^–/–^ mice during the acute infection period was therefore evaluated (Fig. 6). All the mice were infected with 2 × 10^4^ pfu of TMEV to compare the data with the published findings in TNFR1^–/–^ mice. Although the numbers of infected mice that developed acute behavioral seizures were similar for WT and TNFα^–/–^ (67% seized mice, *n* = 18), seizure frequency and severity, as measured by average number of seizures and average cumulative seizure burden, were significantly reduced in TNFα^–/–^ mice (Fig. 6*A*). Interestingly, TNFR2^–/–^ mice experienced severe seizures compared with WT as evidenced by an increase in the number of seizures (WT, 2.8 ± 0.42, *n* = 27; TNF2^–/–^, 4.6 ± 0.43, *n* = 30; *p* = 0.0042) and cumulative seizure burden at 7 and 8 dpi (Fig. 6*B*). The latency to develop the first seizure was also significantly reduced in TNFR2^–/–^, as 11 of 30 TNFR2^–/–^ mice experienced seizures at 3 dpi compared with just 1 of 27 WT mice. In addition, the overall percentage of seizure free-mice for TNFR2^–/–^ and WT mice over the entire acute seizure period was significantly different (*p* = 0.0046). Because previous work demonstrated that TNFR1^–/–^ mice were less susceptible to developing acute seizures (Kirkman et al., 2010) and TNFR2^–/–^ mice developed severe TMEV-induced seizures, we reasoned that TNFR1^–/–^TNFR2^–/–^ mice might have seizure patterns similar to those of WT mice. However, TNFR1^–/–^TNFR2^–/–^ mice exhibited a significantly reduced average number of seizures (WT, 3.1 ± 0.50, *n* = 28; TNFR1^–/–^TNF2^–/–^, 1.0 ± 0.34, *n* = 30; *p* = 0.001) as well as average cumulative seizure burden at 6, 7, and 8 dpi (Fig. 6*C*) compared with WT mice. The percentages of total numbers of infected mice that developed acute behavioral seizures in TNFR1^–/–^, TNFR1^–/–^TNFR2^–/–^, TNFα^–/–^, WT, and TNFR2^–/–^ were 10% (*n* = 2/20; Kirkman et al., 2010), 33% (*n* = 10/30), 67% (*n* = 12/18), 73% (*n* = 40/55), and 93% (*n* = 28/30), respectively (Fig. 6*D*). Handling-induced seizures or spontaneous seizures were not observed in any of the naive or PBS-injected mice from these strains of genetically modified mice. Both male and female mice had similar seizure responses in all the strains tested, and thus, the data from both sexes are pooled. In contrast to the lack of seizure control observed with XPro1595 treatment, results in the genetically modified mice strains suggest that TNFα signaling through the TNFR1 pathway could indeed be a prominent mechanism through which hyperexcitability and seizure activity occur after TMEV infection. In addition, TNFα signaling through the TNFR2 pathway, as is the case in other neurologic disorders, may be involved in dampening excitability, since mice lacking TNFR2 have a higher incidence and greater severity of seizures.

### Increase in the surface levels of AMPAR subunits during acute seizures in WT B6 mice

**Figure 7.**
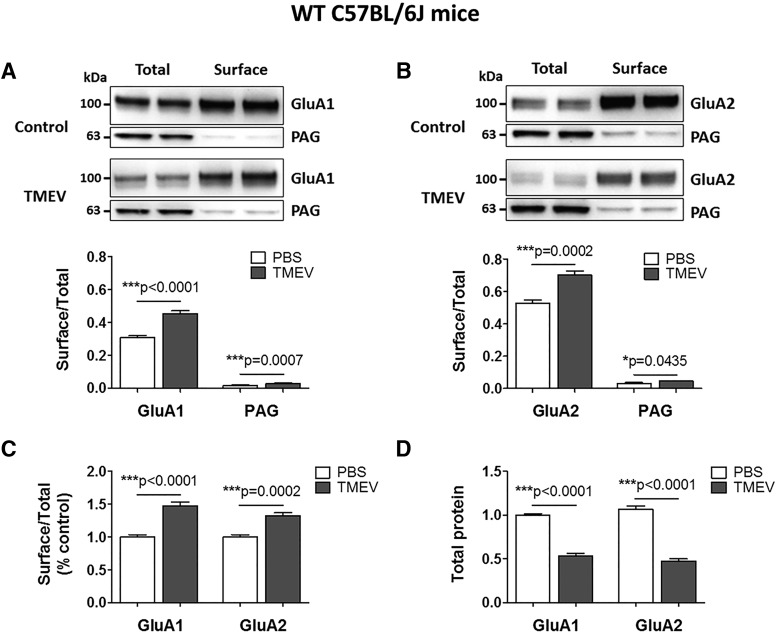
Increase in the cell-surface levels of GluA1 and GluA2 subunits of AMPARs in TMEV-infected WT C57BL/6J mice during acute seizures. (A) Representative immunoblots from two mice show the levels of GluA1 in the total as well as the cell-surface fractions of proteins isolated from ipsilateral hippocampus at 5 d postinjection of PBS (control) or TMEV. Data in the first (total) and the third (surface) lanes from the left are from the same mouse, and the second (total) and the fourth (surface) lanes correspond to the other mouse. The surface proteins were isolated from the intracellular proteins by cell-surface biotinylation in acute hippocampal slices. Levels of GluA1 were quantified by densitometry, and data are shown as ratio of surface to total protein, which is significantly increased in TMEV-infected mice (*n* = 6). PAG is a mitochondrial protein and serves as an intracellular control protein. (B) Similar to GluA1, the ratio of surface/total level for GluA2 is also increased in TMEV-infected mice (*n* = 6). (C) Ratios of surface/total protein expression for GluA1 and GluA2 are increased by 48% and 33%, respectively (data normalized to control). (D) Approximately 50% decrease in the total expressions of GluA1 and GluA2 in TMEV-infected mice compared with control group. Statistics: unpaired two-tailed *t* test.

AMPARs are the primary glutamate receptors that mediate fast excitatory neurotransmission in the brain (Traynelis et al., 2010). TNFα has been shown to increase AMPAR trafficking into postsynaptic neuronal membranes via neuronal TNFR1 (Beattie et al., 2002; Stellwagen et al., 2005). In addition, we have previously demonstrated that the amplitudes of mEPSCs are increased during the acute infection period in CA3 pyramidal neurons, suggesting an increase in AMPA receptor surface expression (Smeal et al., 2012). Given the hypothesized role of TNFα in AMPAR trafficking and the observed increase in mEPSC amplitudes after TMEV infection, we evaluated hippocampal AMPA receptor expression using a cell-surface biotinylation assay in mice treated with TMEV. Biotinylated surface proteins were separated from nonbiotinylated intracellular proteins using avidin beads in tissue prepared from acute hippocampal slices obtained from TMEV- and PBS-treated mice at 5 dpi as described (Gabriel et al., 2014). The GluA1 and GluA2 subunits of AMPARs were probed in the SP and TP fractions by Western blot (Fig. 7*A*). The ratios of surface/total levels of GluA1 that indicate the relative levels of GluA1 present on the cell surface were significantly elevated in the TMEV-infected mice with seizures compared with controls (OD: 0.31 ± 0.011 vs. 0.45 ± 0.018, *p* < 0.0001, *n* = 6). We also probed for phosphate-activated glutaminase (PAG), a mitochondrial protein, to control for the biotinylation of intracellular proteins. The levels of PAG in the SP fraction were <5% compared with those in the TP fraction (OD: 0.015 ± 0.0026 in PBS, 0.03 ± 0.0015 in TMEV) indicating that the biotinylation was largely restricted to cell-surface proteins. The surface/total ratio for the levels of GluA2 was also significantly increased in TMEV-infected mice compared with controls (OD: 0.53 ± 0.018 vs. 0.7 ± 0.025, *p* = 0.0007, *n* = 6; Fig. 7*B*). The cell-surface levels of GluA1 and GluA2 subunits were increased by 48% (*p* < 0.0001) and 33% (*p* = 0.0002), respectively, in TMEV-infected mice compared with PBS-injected mice (Fig. 7*C*). The total protein levels of GluA1 and GluA2 in the TMEV-infected mice were significantly reduced by 46% and 55%, respectively, compared with control mice (Fig. 7*D*). Because TMEV-infected mice have a pronounced neuronal loss in the CA1 region, it is not surprising that the total protein levels of GluA1 and GluA2 are decreased in TMEV-infected mice.

**Figure 8.**
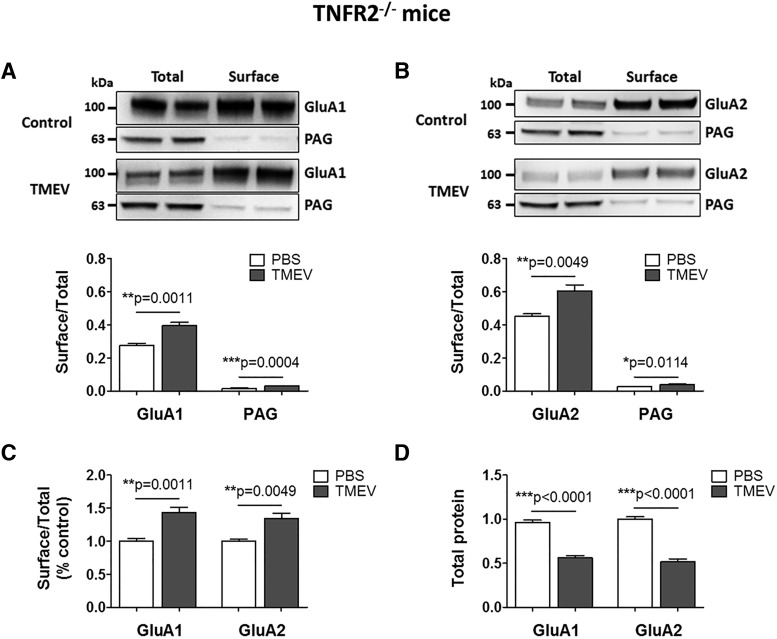
Increase in the cell-surface levels of GluA1 and GluA2 subunits of AMPARs in TMEV-infected TNFR2^–/–^ mice during acute seizures. (A) Representative immunoblots from two mice show the levels of GluA1 in the total as well as the cell-surface fractions of proteins isolated from ipsilateral hippocampus at 5 d postinjection of PBS (control) or TMEV. The surface proteins were isolated by cell-surface biotinylation procedure in acute hippocampal slices. Levels of GluA1 were quantified by densitometry, and data are shown as ratio of surface to total protein that is significantly increased in TMEV-infected mice (*n* = 6). PAG is a mitochondrial protein and serves as an intracellular control protein. (B) Similar to GluA1, the ratio of surface/total level for GluA2 is also increased in TMEV-infected mice (*n* = 6). (C) Ratios of surface/total protein expression for GluA1 and GluA2 are increased by 44% and 34%, respectively (data normalized to control). (D) Approximately 50% decrease in the total expression of GluA1 and GluA2 in TMEV-infected mice compared with control group. Statistics: unpaired two-tailed *t* test.

Because TNFR2^–/–^ mice had severe acute seizures compared with WT mice, we also conducted cell-surface biotinylation assay in TNFR2^–/–^ mice to test whether TNFR2^–/–^ mice had higher cell-surface levels of GluA1 and GluA2 at 5 dpi. Similar to TMEV-infected WT C57BL/6J mice, the ratios of surface/total levels of GluA1 and GluA2 were significantly elevated in the TMEV-infected TNFR2^–/–^ mice with seizures compared with PBS-injected TNFR2^–/–^ mice (OD: GluA1, 0.28 ± 0.012 vs. 0.40 ± 0.021, *p* = 0.0011, *n* = 6; GluA2, 0.45 ± 0.016 vs. 0.61 ± 0.036, *p* = 0.0049, *n* = 6; Fig. 8*A*, *B*). The cell-surface levels of GluA1 and GluA2 subunits were increased by 44% (*p* = 0.0011) and 34% (*p* = 0.0049), respectively, in the TMEV group compared with control mice (Fig. 8*C*). The total protein levels of GluA1 and GluA2 in the TMEV-infected TNFR2^–/–^ mice were significantly reduced by 42% and 48%, respectively, compared with control mice (Fig. 8*D*), likely because of the widespread neurodegeneration observed in CA1 of the hippocampus. The ratios of surface/total levels of GluA1 and GluA2 were not different between TNFR2^–/–^ and WT mice with seizures. It is not clear why TNFR2^–/–^ mice develop severe seizures in response to TMEV infection. However, the effects of TNFα predominantly mediated via TNFR1 in TNFR2^–/–^ mice may decrease seizure threshold by mechanisms other than AMPAR trafficking.

## Discussion

**Figure 9.**
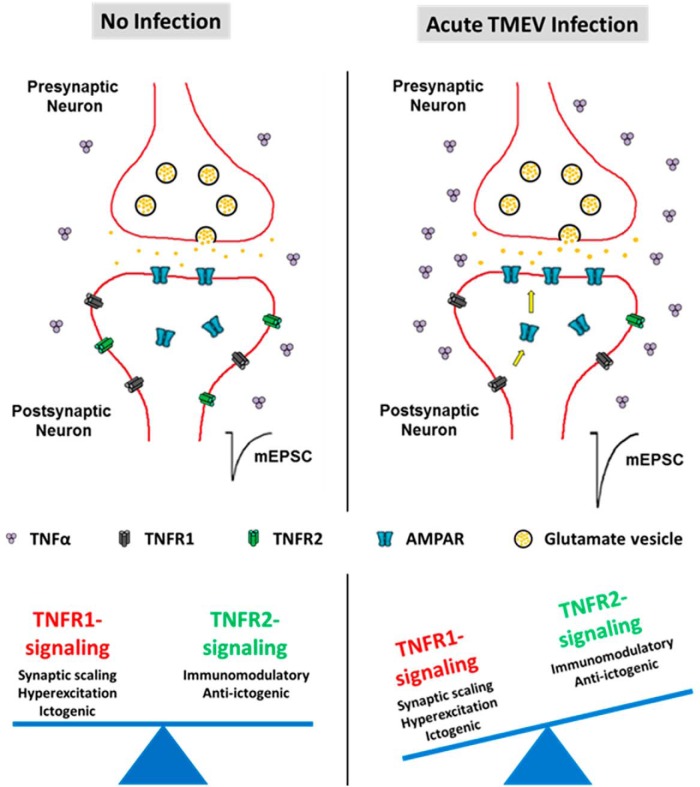
Effects of TNFα mediated predominantly through TNFR1 signaling may contribute to hyperexcitation and the generation of TMEV-induced acute seizures. The protein level of TNFα, the ratio of TNFR1:TNFR2 protein expression, cell-surface to total expression of GluA1 and GluA2 subunits of AMPAR, and excitatory synaptic transmission increase in the hippocampus during the development of acute seizures 3–7 days after TMEV infection in C57BL/6J mice. The results described in this article suggest that the opposite effects of TNFα on neuronal transmission mediated via TNFR1 and TNFR2, with the activation of the TNFR1 pathway causing hyperexcitation by synaptic upscaling of AMPAR, could be implicated in causing seizures after TMEV infection.

Our results suggest that TNFα and its effects mediated via activation of TNFR1, or reduced signaling through TNFR2, could be one of the major inflammatory pathways contributing to TMEV-induced acute seizures (summarized in Fig. 9). We demonstrate here, for the first time, that there is a substantial increase in the protein level of TNFα coincident with an increase in the protein expression ratios of TNFR1:TNFR2 in the hippocampus of TMEV-infected mice with acute seizures. The role of TNFα in contributing to TMEV-induced seizure generation was also supported by our findings in a number of genetically modified mice. We found a decrease in the incidence, frequency, and severity of acute seizures in TNFα^–/–^ and TNFR1^–/–^TNFR2^–/–^ mice, but an increase in frequency and severity of acute seizures in TNFR2^–/–^ mice. Further, consistent with the hypothesized role of TNFα in hippocampal AMPAR trafficking, there is a significant increase in the cell-surface to total AMPAR ratio in TMEV-infected mice with seizures. This increase in cell-surface expression likely underlies the increased amplitudes of mEPSCs that we have previously observed in CA3 pyramidal cells in brain slices from TMEV-infected mice (Smeal et al., 2012). Although peripheral as well as central administration of XPro1595, an inhibitor of sTNFα, failed to inhibit TMEV-induced acute seizures, the data acquired from WT and genetically modified animals suggest that signaling through the TNFα system may play an important role in seizure generation during the acute infection period and may serve as an important therapeutic target in conditions in which inflammation contributes to seizure generation.

TNFα signaling contributes to the physiologic regulation of homeostatic synaptic plasticity by modulating the postsynaptic surface levels of AMPARs (Beattie et al., 2010). TNFα induces synaptic scaling via neuronal TNFR1 by increasing the cell-surface expression of GluA1-containing and GluA2-lacking AMPARs in cultured hippocampal neurons (Beattie et al., 2002; Stellwagen et al., 2005), in the rat hippocampal slice preparation (Stellwagen et al., 2005; Stellwagen and Malenka, 2006), in an animal model of SCI (Ferguson et al., 2008), and in the spinal cord neurons in a model of inflammatory pain (Choi et al., 2010). Elevated levels of AMPARs in postsynaptic membranes can increase the strength of excitatory synaptic transmission. Furthermore, GluA2-lacking AMPARs are Ca^2+^-permeable and increased intracellular concentration of Ca^2+^ can result in excitotoxicity (Liu and Zukin, 2007). We report that the levels of GluA1 and GluA2 subunits of AMPARs on the cell surface are elevated in the hippocampus of mice with acute seizures compared with noninfected mice. Differences in the trafficking of GluA2 subunits between SCI, pain models, and the present study may reflect the differences in underlying pathologic, anatomic, and experimental conditions. Indeed, TNFα has been shown to exert varying effects on the regulation of excitatory synaptic strength contingent on factors such as brain region. TNFα drives the internalization of GluA1 and GluA2 subunits of AMPARs in medium spiny neurons in the striatum, which is in contrast to its effects in hippocampal neurons (Lewitus et al., 2014).

The data acquired here suggests that the increased cell-surface expression of AMPAR subunits most likely occurs in CA3 pyramidal neurons for the following reasons. First, AMPA receptors are expressed in principal cells of the hippocampus (Osten et al., 2006). Second, CA1 pyramidal neurons start to degenerate by 3 dpi and exhibit extensive loss by 4 dpi (Loewen et al., 2016). Third, significant increases in amplitude of mEPSCs occur in CA3 pyramidal neurons in TMEV-infected mice with seizures between 3 and 7 dpi (Smeal et al., 2012). Finally, although the DG is frequently involved in seizure generation in limbic epilepsy models (Sloviter et al., 2012) and TNFα and TNFRs regulate homeostatic synaptic plasticity by enhancing excitatory synaptic strength in the DGCs in response to denervation-induced injury (Becker et al., 2013, 2015), our results show no change in the amplitude of mEPSCs recorded from DGCs in TMEV-infected mice with seizures between 3 and 7 dpi. The DG is relatively intact after TMEV infection, as opposed to CA1 and CA3 regions. The differential susceptibility of various regions of hippocampus to TMEV-induced neuronal damage may also reflect diverse region-specific biochemical and physiologic changes after TMEV infection. It is entirely possible that the varying expression levels of TNFα and TNFRs in different parts of hippocampus may affect the AMPA receptor trafficking differently, which could be investigated in the future. It is important to mention that AMPARs are also present in glial cells, although with much less density than in neurons (Traynelis et al., 2010), and hippocampal interneurons express mainly GluA1 and GluA4 but lack GluA2 (Osten et al., 2006); therefore, we do not rule out the possibility of some postsynaptic AMPAR dynamics in these cells. A novel approach for real-time *in vivo* monitoring of AMPAR subunits has recently been described (Zhang et al., 2015). This approach could be used in future studies to address the question of spatial and cellular targeting of AMPAR trafficking in the hippocampus after TMEV infection.

The time course of changes in the protein and mRNA levels of TNFα, TNFR1, and TNFR2 in the hippocampus is correlated with the development of acute behavioral seizures in the TMEV model. However, our data do not establish the causality between an increase in the TNFα-TNFR1 signaling and the occurrence of acute seizures. Whether the changes in the levels of TNFα and TNFRs at 4–5 dpi are consequent to seizures or viral infection itself, or the seizures are precipitated after an increase in TNFα-TNFR1 signaling or decrease in TNFα-TNFR2 signaling is not clearly understood. Nevertheless, TNFR1^–/–^ and TNFR1^–/–^TNFR2^–/–^ mice were found to be highly resistant to developing TMEV-induced acute seizures, and those with seizures had much less severe seizures compared with WT mice (Kirkman et al., 2010). In addition, although the percentage of TMEV-infected TNFα^–/–^ mice developing acute seizures was similar to that of WT mice, frequency and severity were significantly reduced in TNFα^–/–^ mice. Taking these findings together with the AMPAR trafficking findings, we conclude that TNFα is likely implicated in synaptic scaling via TNFR1, resulting in hippocampal hyperexcitability and seizures. Future studies should investigate molecular mechanisms to test the cause-and-effect relationship between TNFα signaling and the development of acute seizures in this model.

Inhibition of sTNFα-TNFR1–mediated effects and sparing tmTNFα-TNFR2–mediated effects of TNFα has been a well-rationalized strategy to treat many peripheral and CNS inflammatory conditions, and also minimizes adverse effects associated with absolute inhibition of TNFα (Fischer et al., 2015). In previous studies, the concentration of XPro1595 in CSF and plasma were found to be 1–6 ng/ml and 1–8 µg/ml, respectively, after subcutaneous treatment of rats with 10 mg/kg XPro1595 (Barnum et al., 2014). A 10-fold higher level of XPro1595, as used here, would be expected to exchange 99% of endogenous sTNFα (Steed et al., 2003). In the present studies, the average concentration of TNFα in the hippocampus was 110.5 pg/ml at 5 dpi in mice with acute seizures. Thus, if the CSF concentration of XPro1595 in rats after 10 mg/kg s.c. dosing could be extrapolated to TMEV-infected mice, 10 mg/kg s.c. dosing should have been sufficient to remove the endogenously active form of sTNF. That XPro1595 treatment was unable to influence TMEV-induced seizures could be due to an insufficient concentration of XPro1595 in the hippocampus. This could also have been the limitation in a recent study in which only CNS, but not peripheral, administration of XPro1595 had beneficial effects in the mouse model of SCI (Novrup et al., 2014). However, i.c.v. infusion of XPro1595 also failed to reduce TMEV-induced seizures in the present study. Further data on the pharmacokinetics of XPro1595 in TMEV-infected mice could facilitate a better design of dosing regimen for treatment with XPro1595. However, even if XPro1595 sufficiently removed endogenous sTNFα, TNFR1 may still be activated by tmTNFα. Although sTNFα is a major ligand for TNFR1, tmTNFα has been shown to induce inflammation and cytotoxicity through TNFR1 (Horiuchi et al., 2010). A recent study showed that tmTNFα was sufficient to cause inflammation in a gout model, and therefore, inhibition of sTNFα by XPro1595 did not provide beneficial effects (Amaral et al., 2016). Moreover, lymphotoxin (LTα3), formerly known as TNFβ, binds to both TNFRs (Tracey et al., 2008). LTα3-TNFR1 signaling can cause inflammation similar to sTNFα-TNFR1 effects, which could partly explain the lack of effects of XPro1595 on TMEV-induced seizures. Several TNFR1-specific inhibitors are under development (Fischer et al., 2015), which should be leveraged to test the hypothesis of a pathogenic role of TNFR1-mediated signaling in TMEV-induced seizures. Finally, it has been found in previous studies that IL-6 signaling can also contribute to TMEV-induced acute seizures (Kirkman et al., 2010; Libbey et al., 2011). Animals deficient in IL-6, or treated with wogonin to reduce infiltrating macrophages, which are the major source of IL-6 (Cusick et al., 2013), also have a decrease in acute seizure activity in this model (Kirkman et al., 2010). Therefore a polytherapy approach involving both TNF and IL-6 modulators may prove the most efficacious in preventing TMEV-induced seizures.

Similar to the TMEV model, diametric roles of TNFRs in modulating seizure activity have been reported in kainic acid and kindling models of limbic seizures (Weinberg et al., 2013). Our results also corroborate clinical findings in which the levels of TNFR1 and TNFR1-signaling proteins are increased in resected hippocampal tissues from patients with refractory TLE (Yamamoto et al., 2006). Although animal studies that investigate the role of the TNFα system in various models of limbic seizures suggest that TNFR1-mediated signaling contributes to ictogenic effects, the consequences of pathogenic levels of TNFα on seizures are highly context dependent. For example, overexpression of murine TNFα in astrocytes decreased kainate-induced seizures (Balosso et al., 2005), whereas overexpression of murine TNFα in neurons either caused seizures (Probert et al., 1995) or had no effects on kainate-induced seizure activity (Weinberg et al., 2013). Multiple factors in the CNS including the source and the type of TNFα, the cell types expressing TNFRs, levels of TNFα, and relative density of TNFRs in the tissue can influence the seizure outcome (Probert, 2015). These factors must be investigated in detail by designing animal studies that reflect clinical findings from the patients with TLE.

In conclusion, we have demonstrated that pathogenic levels of TNFα and an increase in the expression ratio of TNFR1:TNFR2 in the hippocampus are associated with acute behavioral as well as focal hippocampal electrographic seizures in TMEV-infected mice. TNFα might cause hyperexcitation in TMEV-infected mice by strengthening excitatory synapses via a TNFR1-mediated mechanism, whereas TNFR2 may provide anti-ictogenic effects during acute infection. Anti-TNFα antibodies are widely prescribed for peripheral inflammatory conditions; however, they also cause serious adverse effects by inhibiting desirable functions of TNFα (Traynelis et al., 2010). Therefore, TNFR1-specific inhibitors or TNFR2-specific agonists, as stand-alone therapy or in combination with antiseizure medications, might be a better strategy for reducing seizures after CNS infection, and the disease modifying potential of such compounds should be evaluated in future studies.

## Synthesis

Reviewing Editor: Quentin Pittman, University of Calgary Decisions are customarily a result of the Reviewing Editor and the peer reviewers coming together and discussing their recommendations until a consensus is reached. When revisions are invited, a fact-based synthesis statement explaining their decision and outlining what is needed to prepare a revision will be listed below. Note: If this manuscript was transferred from JNeurosci and a decision was made to accept the manuscript without peer review, a brief statement to this effect will instead be what is listed below.

The response to the reviewers is adequate. At the proof stage please ensure that the reference Gabriel et al ( line740) is complete.

Although a response is not required, the authors should note that increased brain excitability mediated by TNF has been recently reported to involve increased numbers of Glur2 AMPA receptors in the membrane ( Riazi...Pittman, JNS 2015
